# P-624. Changes in Pneumoccocal serotypes in adults and children after 2 years of introduction of PCV13 in National Inmunization Plan in Colombia

**DOI:** 10.1093/ofid/ofae631.822

**Published:** 2025-01-29

**Authors:** Omar Huang Escobar Franco, Erika Alejandra Giraldo

**Affiliations:** Universidad CES, Bogotá, Distrito Capital de Bogota, Colombia; Universidad CES, Bogotá, Distrito Capital de Bogota, Colombia

## Abstract

**Background:**

Pneumococcal disease remains a significant public health concern globally, particularly affecting vulnerable populations such as children and adults. The introduction of pneumococcal conjugate vaccine 13 (PCV13) into national immunization plans has aimed to reduce the burden of pneumococcal disease by targeting specific serotypes. However, monitoring changes in pneumococcal serotypes following vaccine introduction is crucial for understanding vaccine effectiveness and potential serotype replacement.

Distribution of Streptococcus pneumoniae serotype isolates by age in Colombia 2023

**Figure:**
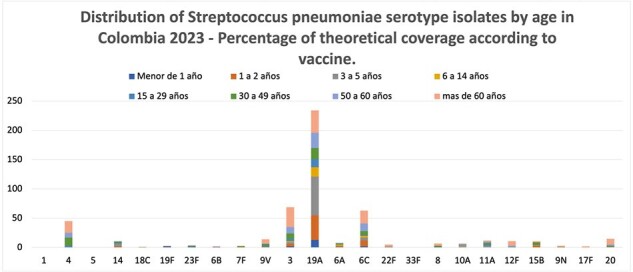

Distribution of Streptococcus pneumoniae serotype isolates by age in Colombia by age group, INS data as of December 2023.

**Methods:**

In this study, data were extracted from the National Institute of Health for the Surveillance of Pneumococcus in Colombia. We conducted a comparison of proportions to analyze changes in pneumococcal serotypes among both adults and children after two years of PCV13 introduction. Serotype distribution was examined pre- and post-vaccine introduction to identify shifts in prevalence.

Distribution of Streptococcus pneumoniae serotype isolates in children under 5 years of age, Colombia 2023

**Figure:**
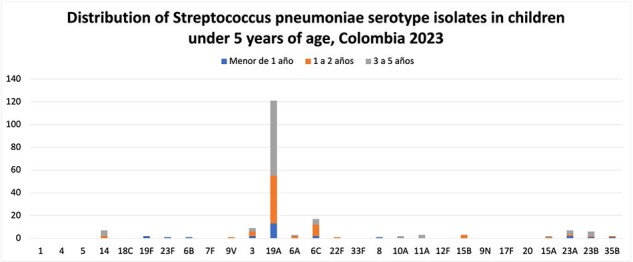

Distribution of Streptococcus pneumoniae serotype isolates in children under 5 years of age in Colombia by age group, INS data as of December 2023.

**Results:**

Our analysis revealed a notable decrease in vaccine serotypes among both adults and children following the introduction of PCV13. However, an increase in non-vaccine serotypes was observed during the same period. Of particular significance, isolates of serotypes 19A, 3, and 6C continued to predominate, suggesting a persistent challenge in combating these serotypes despite vaccination efforts.

Distribution of Streptococcus pneumoniae serotype isolates in persons over 60 years old, Colombia 2023

**Figure:**
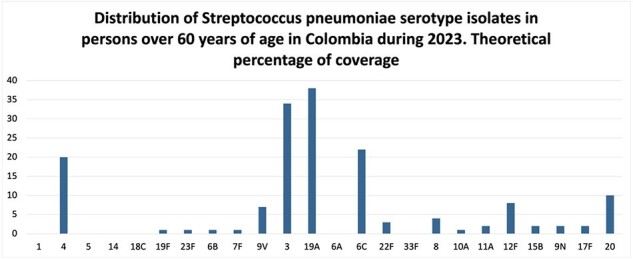

**Conclusion:**

The findings of this study underscore the complex dynamics of pneumococcal serotype distribution following the introduction of PCV13. While the vaccine has led to a reduction in vaccine-targeted serotypes, the emergence of non-vaccine serotypes presents a new challenge for pneumococcal disease prevention and control efforts. Continued surveillance and research are essential to monitor serotype trends and inform vaccine strategies to effectively combat pneumococcal disease in both children and adults.

**Disclosures:**

**Omar Huang Escobar Franco, MD, MSc, PhD.**, Pfizer: Honoraria

